# Interface Characterization of Consolidated PPGF Tapes on PPGF Mat Material

**DOI:** 10.3390/polym15040935

**Published:** 2023-02-14

**Authors:** Andreas Kapshammer, Matei Constantin Miron, Lukas Dangl, Zoltan Major

**Affiliations:** 1 Institute of Polymer Product Engineering, Altenberger Straße 69, 4040 Linz, Austria; 2 Competence Center CHASE GmbH, Altenberger Straße 69, 4040 Linz, Austria; 3 Greiner Perfoam GmbH—Eine Gesellschaft der NEVEON Gruppe, Mainstraße 1, 4470 Enns, Austria

**Keywords:** single ply peel test, mandrel peel test, strain energy release rate, orthotropic PPGF mat material, unidirectional thermoplastic tapes

## Abstract

Laminated composites with thermoset matrices are already well established in major engineering fields like automotive and aviation. The primary drawbacks of such thermoset-based composites are the high cycle times required during manufacturing and their limited potential for recycling. Providing an alternative to thermoset-based composites, thermoplastic matrix materials gained more and more momentum by addressing these previously mentioned drawbacks. The preferred manufacturing technique for these materials employs fiber-reinforced thermoplastic tapes consolidated and formed together with a compatible substrate. The most critical aspect for all these applications is the stress or load transfer between the thermoplastic tapes and the substrate. If the interface is too weak and fails prior to the substrate or tape, a high amount of theoretical mechanical performance is lost. The presented research investigates the influence of variations in manufacturing parameters, within the industrially relevant process window, on the interface strength of the final composite. The investigated composite material consists of PPGF UD tapes consolidated on a PPGF mat substrate. In particular, the influence of the consolidation parameters of pressure, temperature, and time are of special interest. The results of this work reveal a 400% increase in the measured mean strain energy release rate upon increasing the consolidation time from 60 s to 120 s at a consolidation temperature of 230 °C and a pressure of one bar. In contrast to this, an increase in the consolidation pressure, at constant temperature and time, leads to a minor improvement in the *G_C_* value of 20%. For testing and characterizing the corresponding interface properties, a mandrel peel testing setup was employed.

## 1. Introduction

### 1.1. Motivation

Fiber-reinforced composites based on thermoplastic matrix materials, as well as their thermoset-based counterparts, have high potential in the field of lightweight design due to their outstanding specific properties. Thus, this material class is one of the main development areas in the transportation sector, including automotive and aviation. According to the AVK-Handbook of Composites [1], an increasing future potential is observed in all fields where a substitution of metallic structures (steel or aluminum) is beneficial in terms of weight reduction. Thermoplastic matrix materials have some essential advantages in terms of recyclability and can be more easily integrated into a sustainable and circular product life cycle [1,2]. The well-established industrial process of stamp forming is the main processing technique for forming thermoplastic tapes. Thereby, a previously consolidated, two-dimensional plate is deformed into its desired three-dimensional shape by applying pressure and temperature [3]. Additionally, a localized reinforcement of already existing materials (substrate) via the application of single tapes is a common procedure in industrial optimization processes. Therefore, the formed interface between the locally placed tapes and the substrate is responsible for the load transfer. Consequently, the mechanical properties of the materials are crucial for the structural integrity of the final component [4]. The interface formation between two thermoplastic polymer pairs is generally based on inter-diffusion processes in areas of intimate contact [5,6,7].

### 1.2. State of the Art

**Figure 1:**
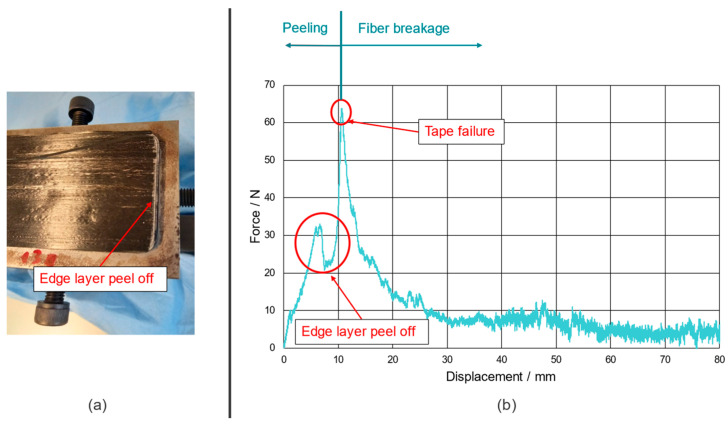
Representative result of preliminary 90° peel tests performed on single PPGF UD tape specimens. (**a**) Failed specimen after initial edge layer peel of due to the bending curvature of the peel arm being too small; (**b**) corresponding force-displacement curve indicating the region of edge layer peel off and tape failure.

Over the last few decades, a variety of different experimental setups were developed to determine the fracture toughness of bonded structures and components. These experiments originate in the field of adhesive joints, and the findings were translated to investigate and analyze composites and sandwich structures [8,9,10,11,12]. Already standardized experimental procedures for interface testing of such structures are the flexible peel arm test combined with a stiff substrate and the 90°, fixed arm, floating roller, and climbing drum peel tests. The former three tests are also described in the DIN EN 1464 and DIN EN 28510-1 standards, whereas the last one is documented in the ASTM D 1781 standard. For specimens including a stiff substrate as well as a stiff peel arm (or for monolithic specimens), classical fracture mechanics methods like the double cantilever beam (DCB) experiment, as specified in the ASTM D 3433, are utilized. When there is a highly compliant peel arm, the tested specimen tends to deform in such a way that the reinforcement will fail due to bending stresses before the failure of the interface. This phenomenon appears, for example, when performing a 90° peel test on a single PPGF UD tape from a PPGF mat material, as we investigated this in our preliminary experiments. A depiction of one of these experiments can be seen in Figure 1, where (a) is displaying the failed specimen and (b) is presenting the corresponding force-displacement curve.

To adress the previously mentioned problem of a failing UD tape prior to interface failure, the so-called mandrel peel test (MPT) was identified as an opportunity to prevent tape failure and experimentally determine the critical mode I strain energy release rate, $G_{IC}$, of the interface [8,13]. The MPT was critically investigated in the work of [8,9,14] to evaluate the applicability and reliability of this measurement method when using different applications and materials. Grouve et al. [9] showed that the fracture toughness values determined using the MPT approach are about 20% lower compared to the ones determined using a DCB test. To explain this, it was stated that the MPT procedure yields to a lower number of fiber bridging effects in the crack zone. Moreover, the linkage between the matrix and fibers is crucial regarding failure and breakage mechnisms within the composite. Marcuello et al. [15] and Berzin et al. [16] investigated the influence of different matrix/fiber combinations focused on the resulting adhesion force based on the molecular interactions of the constituents. Their research revealed strong correlations between the matrix/fiber affinity and the mechanical performance of a derived structure. Due to the presented results of [9], it can be concluded that the MPT shows high potential for providing reliable data in the field of characterizing the interface properites of UD-reinforced thermoplastic composites. Therefore, this study deals with the characterization of the interface properties between PPGF UD tapes and PPGF random mat materials, utilizing a specially designed MPT. Furthermore, the influence of consolidation temperature, pressure, and time on the critical strain energy release rate were determined and quantified.

## 2. Materials and Methods

In the following section, a detailed description of the utilized tape and substrate materials as well as all information concerning the methodology and the experimental setup will be provided.

### 2.1. Materials Used

This study focuses on fiber-reinforced thermoplastic composites consisting of polypropylene (PP) as the matrix material and glass fibers (GF) as the reinforcing phase. In particular, a random glass fiber mat impregnated by the PP matrix forms the substrate which was functionalized with unidirectional (UD) tapes, under the tradename *Gurit Plytron GN 638 T*, based on the same constituents. Both the semi-finished mat and tape materials were provided in a wound-up form on two rolls. The PPGF mat coil had a width of 1.5 m and a total length of 10 m. The corresponding dimensions for the PPGF UD tape coil were 300 mm and 3 m. To manage the introduction of a predefined initial crack, common aluminum foil with a thickness of 0.6 mm was used.

The in-plane mechanical properties of the PPGF mat materials are typically assumed to be isotropic in literature [2,17,18]. Nevertheless, the performed tensile tests in roll direction (IR), normal to roll direction (NR), and at an angle of 45° revealed that the PPGF mat material used shows a non-neglectable amount of anisotropic behavior. The results of these tensile tests, including the corresponding evaluation, will be provided in Section 3.1. Concerning the PPGF UD tape with a glass fiber content of 60 w%, the mechanical properties provided by the material supplier are listed in Table 1.

### 2.2. Methodology and Setup

In this section, the characterization method for the substrate material will be shown, followed by a detailed description of the preparation process for obtaining proper peel test specimens.

#### 2.2.1. Tensile Tests of the PPGF Mat Material

**Figure 2:**
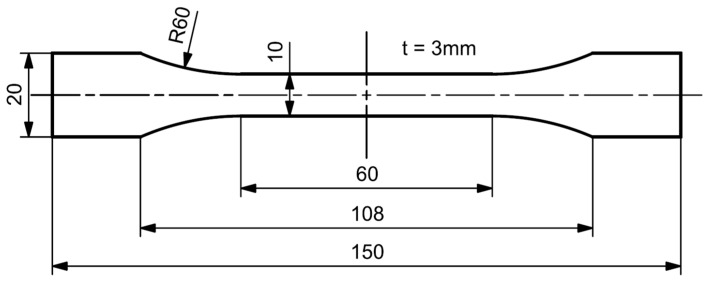
Specimen dimensions according to DIN EN ISO 527-4, type 1B.

For the purpose of characterizing the PPGF mat material, standardized tensile tests as specified by DIN EN ISO 527-4 were performed on the hydraulic testing machine MTS 852 Test Damper System (MTS Systems Corporation, Eden Prairie, MN, USA). A 10 kN load cell was utilized to conduct the experiments and the specimens were clamped using a hydraulic clamping system. To provide an accurate and contactless strain measurement, the digital image correlation (DIC) system ARAMIS 4M (Carl Zeiss GOM Metrology GmbH, Braunschweig, Germany) was employed and used for the evaluation of the results afterwards. The specimen dimensions related to the quoted standard are shown in Figure 2. A portal milling machine was used to mill the specimens out of consolidated plates with in-plane dimensions of 400 × 300 mm^2^ and a thickness of 3 mm. As the purpose of these tests is to investigate the influence of the material’s orientation, the specimens were aligned in roll direction (IR), normal to roll direction (NR), and at an angle of 45°. In each direction, five specimens were tested, and according to the data reduction process an averaged curve was derived. The tensile tests were performed with a velocity of 1 mm/min until specimen failure.

#### 2.2.2. Mandrel Peel Test Specimen

Starting from a processing point of view, the interface between two semi-finished products based on thermoplastic matrix materials has to be established by applying temperature and pressure over a certain amount of time to allow inter-diffusion between the two regions [6,7,19,20,21,22]. Usually, this process step is called consolidation. In this study, a two-stage consolidation process was utilized to manufacture PPGF random mat plates with one PPGF UD tape layer on top. In general, one can distinguish between a heating and cooling stage, each respectively pressed, with different process settings for each. To keep the influencing parameters under control, only the parameters of the heating press were adjusted in terms of temperature, pressure, and time. As an overview, the corresponding process parameters for the heating and cooling presses are summarized in Table 2.

**Figure 3:**
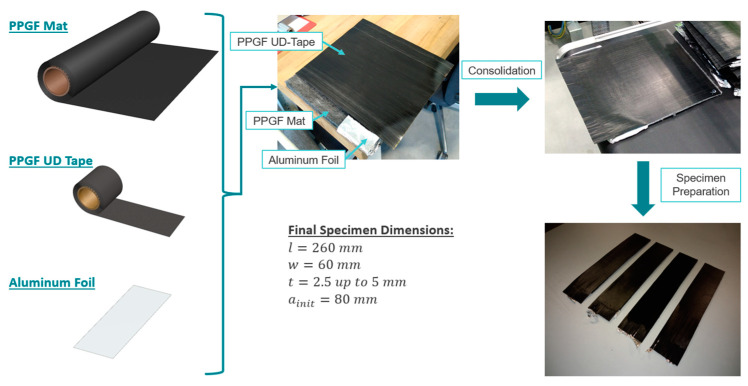
Schematic representation of the specimen manufacturing and preparation workflow.

Previously, the anisotropic material response of the PPGF mat material was mentioned. Thus, the investigation of the relative substrate direction (IR and NR) compared to the UD tape direction is another interesting variable. Obviously, all of the indicated consolidation process parameters for a 60 s consolidation time were used to manufacture two plates for each combination, resulting in 18 consolidated plates in total. For the first plate, the fiber direction of the UD tape was colinear with the NR-direction of the PPGF mat material and vice versa for the second plate. Furthermore, the influence of the consolidation time was analyzed by preparing another four plates for 200 °C and 230 °C with 120 s and 140 s of consolidation time in the NR direction and an applied closing pressure of 1.0 bar. In the following step, four rectangular specimens were cut out of each plate with a thickness of $t_{min}=2.5\;\mathrm{mm}$ up to $t_{max}=5\;\mathrm{mm}$, a length of l=260 mm, and a width of w=60 mm. As a result, 88 specimens for the experimental investigation were prepared. An initial defect of 80 mm length was introduced during the consolidation process by placing an aluminum foil at the interface between the tape and the substrate. The thickness of the plates varies due to differences in the applied consolidation pressures. Figure 3 provides a visual representation of the principal workflow for preparing proper specimens for the mandrel peel test setup, which will be introduced in the next paragraph.

**Figure 4:**
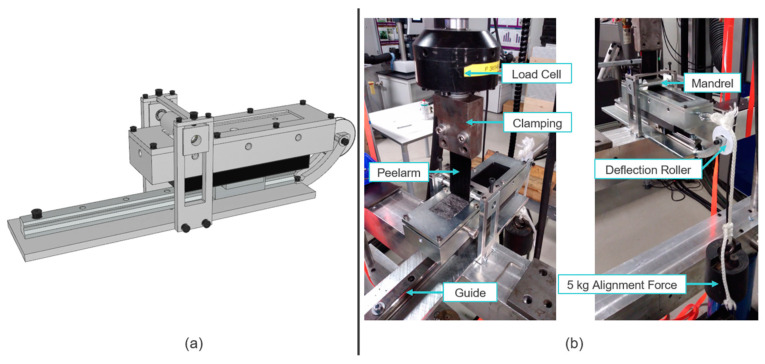
(**a**) Rendering of the mandrel peel test setup; (**b**) Experimental realization of the mandrel peel test setup including specimen, alignment force, and measuring devices.

As initially mentioned in the introduction, a standardized 90° peel test is not applicable when peeling a single UD tape from a certain substrate due to the small bending radius and the subsequent fiber/tape failure. To overcome this issue, the already available peel test setup was further developed into a modular peel test device which can be used for 90° peel tests or it can be easily adapted into a mandrel peel based on the recommendations of [9,13,14]. For performing the physical experiments, the MTS 852 Test Damper System, including a mechanical clamping device and a 10 kN load cell for force data recording, was used. Through the hydraulic actuator, a displacement-controlled measurement with a maximum axial displacement of $u_{ax,\;max}=100\;\mathrm{mm}$ was realized, accomplished at a constant axial velocity of $v_{ax}=1\;\mathrm{mm}/s$. On the left side of Figure 4, a rendering of the mandrel peel test is depicted, and on the right side, the physical setup is depicted.

During the measurement, the axial force, which will be defined as the pulling force, $F_{p}$, and the axial displacement, u, were recorded. According to the suggested evaluation and data reduction method described in [9] and [13], the critical strain energy release rate, $G_{C}$, over u was calculated by the following formula (1):

(1) $$G_{c}=\frac{1}{w}\;\left(F_{p}\left(1-\mu \right)-F_{a}\right)$$

where w=44 mm is defined as the peel arm width, $F_{a}$ is referred to as the alignment force, which in this study is always constant at a value of 5 kg, and μ is the coefficient of friction for the whole system. Based on preliminary investigations, μ was defined with a value of *μ* = 0.27

## 3. Results

In the first part of this section, the experimentally determined results of the tensile tests for the characterization of the PPGF mat substrate material will be presented, followed by the results obtained from the mandrel peel test.

### 3.1. Tensile Tests of the PPGF Mat Material

**Figure 5:**
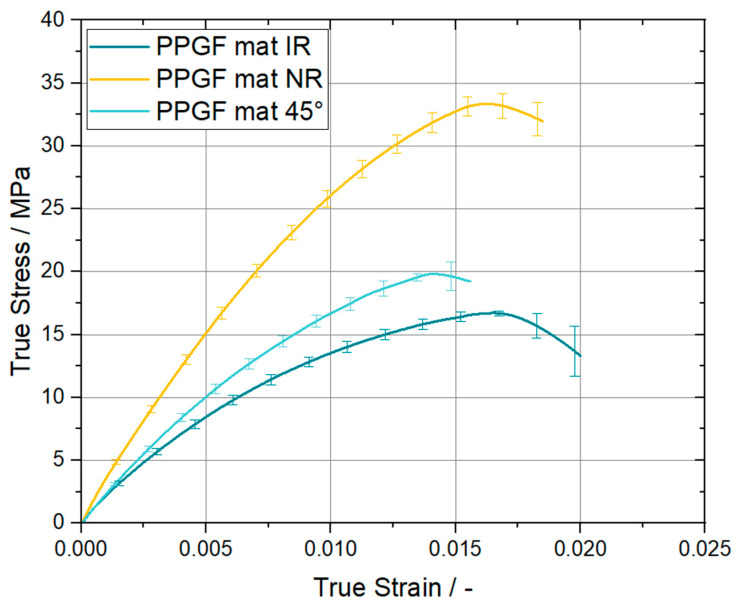
Resulting true stress vs. true strain curves of the investigated PPGF mat material under different orientations with regard to the roll direction (IR—in roll direction, NR—normal to roll direction).

In order to ensure that our tested substrate can be treated as an isotropic material as well ([2,17,18]), we performed standardized tests for different material orientations. In Section 2.2, the setup used as well as the evaluation method were introduced. The true stress vs. true strain curves derived from the tensile experiments are shown in the provided line plot in Figure 5.

The posed data clearly state that the investigated PPGF mat material provides different mechanical properties dependent on the loading direction relative to the roll direction. As indicated by the yellow curve of Figure 5, the mechanical response of specimens oriented in the NR direction was stiffer than the ones oriented in roll direction, which are represented by the dark green line. As expected, the 45° specimens were between those two extrema, with a trend of performance closer to that of the IR specimens. For comparison, the Young’s modulus and Poisson ratio of all three configurations are summarized in Table 3. The former was evaluated according to the standard DIN EN ISO 527-4 and the latter was directly derived from the DIC strain measurement.

### 3.2. Mandrel Peel Test

**Figure 6:**
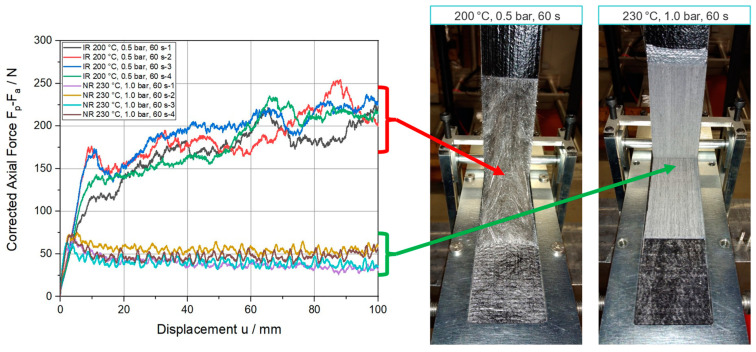
Representative corrected force-displacement curves for the processing settings of 200 °C, 0.5 bar, 60 s (IR) and 230 °C, 1.0 bar, 60 s (NR), including the corresponding failure representations of the specimens.

Based on the recorded data obtained from the measurement setup introduced in Section 2.2, force-displacement curves were defined. In these curves, the corrected axial force, which is the measured axial force, $F_{p}$, subtracted by the alignment force, $F_{a}$, is plotted against the measured axial displacement, u. Some selected examples of such plots are depicted on the left side of Figure 6.

For a proper analysis of the interface properties, a defined and controlled separation between the tape and the substrate is essential. While conducting the experiments, two very different failure patterns were observed. In one instance, the tape was peeled off the substrate due to a specific failure propagation within the interface region of the two materials. Accordingly, the two materials separated due to a controlled failure of the interface, whereas the integrity of the tape and substrate was still present, and the mechanical work put into the system can be considered for the interface characterization. In the other instance, fibers were pulled out of the substrate during the experiments. In such a case, the mechanical work cannot be unequivocally assigned to the interface. Furthermore, this failure is not restricted to the interface only. Therefore, an accurate computation of the strain energy release rate of the interface was not possible due to the involvement of the substrate.

Our test results show that the failure behavior of the specimens consolidated at 200 °C and 230 °C with 0.1 bars of pressure was accompanied by fiber pullout events, rendering the measurement of the interface strain release rate inaccurate. Thus, those experimental results will not be taken into consideration for the interfacial strain release rate calculation.

**Figure 7:**
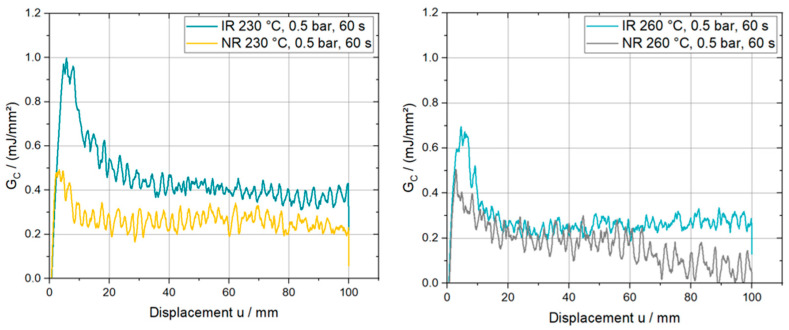
Characteristic GC curves at 230 °C and 260 °C for different roll directions.

For an evaluation of the experiments from a fracture mechanics point of view, the critical strain energy release rate, $G_{C}$, can be calculated with relation (1) for each configuration. In general, the resulting development of $G_{C}$ over the axial displacement, u, indicates a linear region at the beginning until a peak value is reached. This is equivalent to the onset of crack growth starting from the crack tip of the initially introduced crack. Afterwards, the critical strain energy release rate decreases slowly but steadily to a plateau region. A mean value for $G_{C}$ can be calculated by averaging the apparent data points of the plateau. Four characteristic curves are shown in Figure 7 to further investigate the influence of the roll direction on the calculated strain energy release rate.

**Figure 8:**
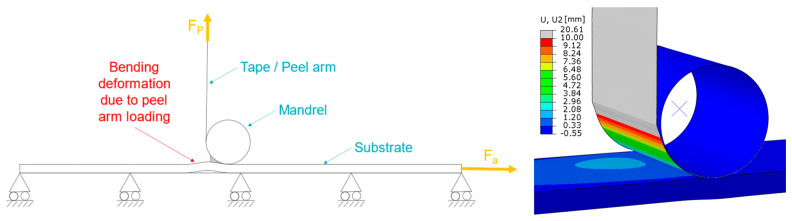
Schematic (**left**) and numerically analyzed (**right**) representation of the substrate bending deformation due to the peel arm loading for compliant substrates.

An equality of the initial slope can be observed at 230 °C and 260 °C, although unexpectedly higher $G_{C}$ values in roll direction (IR) can be seen within the first 10 mm of displacement, as well as a decreasing trend for this effect at higher temperatures. Supported by the previously presented tensile data for the PPGF mat material, local bending deformations of the substrate due to the loading in the peel arm might introduce additional bending energy. Consequently, the apparent $G_{C}$ is overestimated due to the superposition of the energy going into the interface and the energy needed to deform the substrate. For highly compliant substrate materials, a defined and correct derivation of the interface properties using the mentioned evaluation method will not be valid. Thus, the importance of correctly chosen substrate dimensions and materials, is an important finding for future experiments. Furthermore, this bending deformation, which was also experimentally observed, is schematically depicted in Figure 8 together with the corresponding deformation predicted by a finite element simulation using the commercial software package Abaqus FEA (Simulia—Dassault Systemes, Providence, RO, USA). The numerical model consists of a substrate, a tape, and a mandrel region. The substrate and the tape regions interact by means of cohesive contact formulation, while the interaction between the tape and the mandrel region is defined as frictionless, hard contact. The dimensions of the three-dimensional model, as well as the size of the cohesive contact interaction zone, correspond to the manufactured specimen dimensions presented in Section 2.2. Load introduction was performed using imposed displacement boundary conditions, scaled linearly during an implicit general static step. The step time was equal to the experimental time. Fully integrated linear hexahedral elements (C3D8) were preferred over the reduced integrated elements (C3D8R) due to hourglassing effects. In total, 17,160 C3D8 elements were used to model the substrate region, resulting in six elements over the thickness of the substrate (element size 1 mm × 1 mm × 0.41 mm). The tape region was discretized using 6560 fully integrated shell elements (S4) with five integration points over the entire thickness (element size 1 mm × 1 mm). The mandrel region was discretized using 3690 discrete rigid elements (R3D4; element size 1 mm × 1 mm). From a material modelling perspective, the tape is represented by a linear elastic lamina formulation utilizing the parameters presented in Table 1. Further, an anisotropic elasto-plastic material model was used for the substrate, derived from the experimental true stress/strain curves of Figure 5. For interface modelling, an energy-based cohesive contact formulation was applied using the experimentally determined parameters.

**Figure 9:**
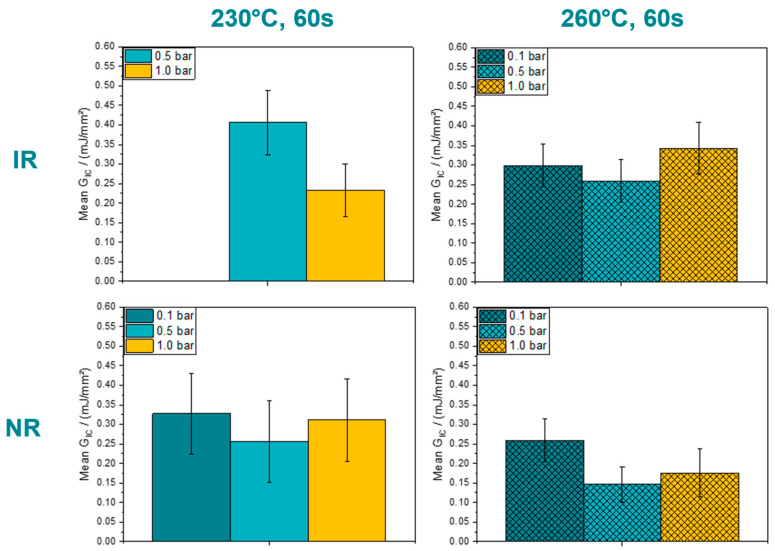
Mean G_C_ values for consolidated PPGF UD tapes on PPGF mat material for various consolidation process settings and substrate orientations at a consolidation time of 60 s.

To compare and evaluate the different process settings, the mean $G_{C}$ values of the plateau region will be considered in the plots below. The bar charts in Figure 9 provide the averaged data according to all evaluable process settings for the two temperatures of 230 °C and 260 °C, three consolidation pressures of 0.1 bars, 0.5 bars, and 1.0 bar, analyzed in and normal to the roll direction of the underlying PPGF mat substrate for a consolidation time of 60 s. In the first plot, where the results for 230 °C in roll direction are provided, the value for a corresponding consolidation pressure of 0.1 bars is missing, caused by cohesive failure of the substrate.

Based on the provided data, a reduction of the mean $G_{C}$ at 260 °C compared to 230 °C can be observed for the setting where the tape is oriented normal to the roll direction of the substrate (NR). In numbers, this decrease can be described as −21%, −43%, and −44% for the corresponding consolidation pressures at 0.1 bars, 0.5 bars, and 1.0 bar. Comparing the results related to the material direction of the substrate, it is again evident that the values for IR are higher than those for NR. The phenomena causing this unexpected behavior was already shown and explained in the discussion Figure 7 and Figure 8. Excluding the first bar chart in Figure 9, an upcoming trend indicating a local minimum at a consolidation pressure of 0.5 bars can be observed. Further, the data set for NR reveals that the maximum obtained value was reached at a consolidation pressure of 0.1 bars by 0.33 mJ/mm² and 0.26 mJ/mm² for 230 °C and 260 °C, respectively. A reverse relationship can be found for the investigated specimens manufactured IR and at 260°C. Furthermore, the significance of consolidation pressure and consolidation temperature were investigated by performing a single factor analysis of variance (ANOVA) at a significance level of 95%. As a result, neither the consolidation pressure nor the consolidation temperature were revealed to have a significant influence on the mean $G_{C}$ value.

**Figure 10:**
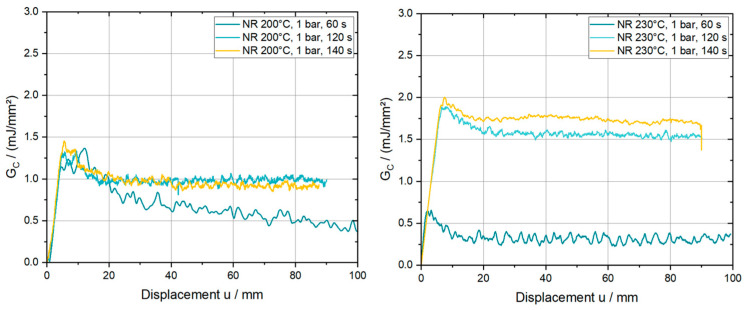
Characteristic G_C_ curves at 200 °C and 230 °C for different consolidation times at a consolidation pressure of 1 bar and in the NR direction.

Besides the parameters of substrate direction, consolidation temperature, and pressure, the consolidation time is also crucial for the resulting interface properties. According to the already mentioned process settings in Section 2.2, the influence of the consolidation time was investigated at 200 °C and 230 °C. For data reduction and evaluation, the equivalent scheme, as already presented, was applied. In Figure 10, the characteristic $G_{C}$ over u curves are plotted for the three different consolidation times of 60 s, 120 s and 140 s. The corresponding closing pressure of 1 bar and the orientation in the NR direction was chosen to eliminate the formerly mentioned bending behavior of the substrate.

**Figure 11:**
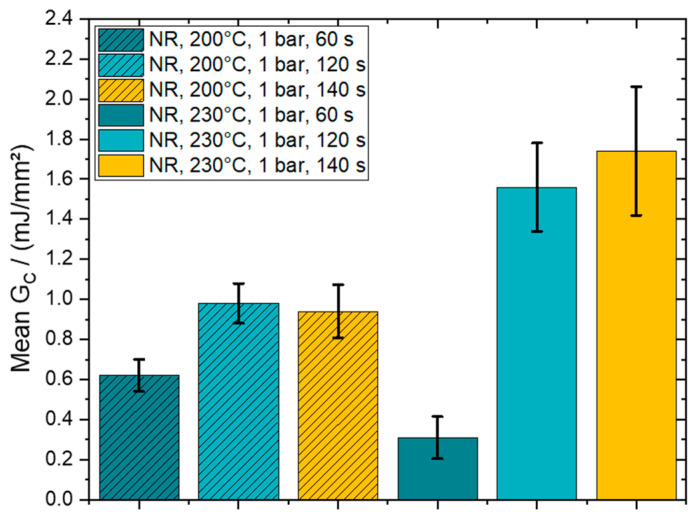
Mean G_C_ values for consolidated PPGF UD tapes on PPGF mat material at 200 °C and 230 °C, 1 bar consolidation pressure and 60 s, 120 s and 140 s consolidation time.

By analyzing the provided plots, one can clearly observe a coinciding initial response for all six line plots, which states that the apparent stiffness of the interface in mode I loading is insensitive across varying consolidation times. However, the damage initiation and plateau value of $G_{C}$ is higher for both temperatures. At 200 °C, the $G_{C}$ plateau values for 120 s and 140 s are on the same level and show a mean increase of 55% compared to the reference values at a consolidation time of 60 s. Further, a jump of 400% and 460% are derived for the curves at 120 s and 140 s, respectively, at a consolidation temperature of 230 °C. The following bar chart in Figure 11 provides the mean $G_{C}$ values, calculated from the plateau region from the results in Figure 9, where the hatched columns represent the results for 200 °C. This figure highlights the high consolidation time dependency, which also reveals a progressive trend for higher temperatures, of the mean strain energy release rate.

From a statistical point of view, again an ANOVA was performed including similar assumptions as before for the analysis regarding consolidation pressure and temperature. Thus, the visually observed impact of the consolidation time is also confirmed as significant in terms of a variance analysis.

## 4. Conclusions and Future Perspectives

In this article, we demonstrated that the mandrel peel test is a proper method for performing peel tests on single thermoplastic tapes consolidated on a substrate. Through the mandrel, a defined bending radius of the tapes can be guaranteed within the experiment, avoiding tape failure prior to interface failure.

Material properties of the PPGF mat material, derived from standardized tensile tests, revealed a non-neglectable amount of anisotropy in the mechanical response. This stands in contrast to the common assumption of isotropic behavior of mat materials in the literature. As a consequence, the influence of the substrate orientation in reference to the tape direction regarding the interface properties was also investigated. As discussed in Section 3.2, higher values for the critical strain energy release rate, $G_{C}$, were derived in the IR direction. Additionally, an observation of the performed measurements showed a certain bending deformation of the substrate introduced by the vertical peel arm loading. Such a response was not visible for the specimens manufactured in the NR direction. Consequently, it was found that $G_{C}$ is overestimated by the superposition of the bending deformation and interface failure in the IR direction compared to the results in the NR direction. Generally speaking, a tendency for a local minimum $G_{C}$ was found at a consolidation pressure of 0.5 bars in association with a general decrease at higher temperatures, whereas neither the first nor the second parameter can be identified as significant from a statistical point of view.

The influence of the consolidation time was investigated by utilizing a process parameter set for plate manufacturing to keep the bending deformation of the resulting substrate as small as possible. The evaluation of these experiments leads to the finding that the initial slope of the characteristic peel curves ($G_{C}$ vs. u) is equal for different consolidation times, whereas the mean strain energy release rate, derived from the plateau region, states a dominant time dependency. $G_{C}$ increases for longer consolidation times and higher temperatures, indicating a progressive trend for the latter parameter. The consolidation time dependency is also evidenced by statistical considerations.

The current study revealed room for improving the accuracy and reliability of the presented measurement method by minimizing the bending deformation of the substrate. This can be achieved by utilizing proper materials or manufacturing settings to generate a stiff enough substrate.

For future research, the theoretical concepts of interface formation, including contact mechanics (intimate contact) and molecular dynamics/diffusion (degree of healing) should be considered. To correlate and validate manufacturing parameters, mechanical properties, and theoretical approaches, the dimensionless degree of bonding can be used [6,7,23]. Due to its definition and multiple dependencies, this quantity is prone to be predicted by the coupling of finite element simulations and neural networks, which will be a main focus in upcoming research activities. However, the gathered knowledge regarding process parameters (consolidation pressure, temperature, and time) on the interface properties of substrate/tape structures can be used in automotive or aerospace applications to further optimize current design or to increase the strength to weight ratio even more [4,24,25,26].

## Figures and Tables

**Table 1:** Mechanical material properties for the Plytron GN 638 T (Data Sheet Values).

Property	Value	Unit
Density *ρ*	1.5	g/cm^3^
Tensile Modulus *E*_11_	28,000	MPa
Tensile Modulus *E*_22_	3720	MPa
Tensile Strength *X*_11_	720	MPa
Strain at Break *ϵ*_max_	1.9	%

**Table 2:** Process parameters for the heating and cooling presses during the consolidation step.

**Process Parameters—Heating Press**	
Temperature/°C	200, 230, 260
Closing Pressure/bar	0.2, 0.5, 1.0
Consolidation Time/s	60, 120 *, 140 *
**Process Parameters—Cooling Press**	
Temperature/°C	40
Closing Pressure/bar	5.0
Consolidation Time/s	60.0

* only for 200 °C and 230 °C, 1 bar closing pressure, and in the normal to roll direction (NR).

**Table 3:** Resulting Young’s modulus and Poisson ratio of the PPGF mat material for different material orientations, derived from standardized tensile tests.

Property	IR	45°	NR	Unit
Young’s Modulus *E*	1900	2100	3100	MPa
(Standard Deviation)	(110)	(210)	(220)	(MPa)
Poisson Ratio *ν*	0.36	0.33	0.31	-

## Data Availability

Data is not publicly available.
